# Extreme phenotypes approach to investigate host genetics and COVID-19 outcomes

**DOI:** 10.1590/1678-4685-GMB-2020-0302

**Published:** 2021-03-01

**Authors:** Michel Satya Naslavsky, Mateus Vidigal, Larissa do Rêgo Barros Matos, Vivian Romanholi Cória, Pedro Benedito Batista, Álvaro Razuk, Paulo Hilário Nascimento Saldiva, Marisa Dolhnikoff, Laire Schidlowski, Carolina Prando, Edécio Cunha-Neto, Antonio Condino-Neto, Maria Rita Passos-Bueno, Mayana Zatz

**Affiliations:** 1 Universidade de São Paulo, Instituto de Biociências, Departamento de Genética e Biologia Evolutiva, Centro de Pesquisa sobre o Genoma Humano e Células-Tronco, São Paulo, SP, Brazil.; 2 Instituto Prevent Senior, São Paulo, SP, Brazil.; 3 Faculdade de Medicina da Universidade de São Paulo (FMUSP), Departamento de Patologia, São Paulo, SP, Brazil.; 4 Instituto de Pesquisa Pelé Pequeno Príncipe, Faculdades Pequeno Príncipe, Hospital Pequeno Príncipe, Curitiba, PR, Brazil.; 5 Hospital das Clínicas da Faculdade de Medicina da Universidade de São Paulo, Instituto do Coração, São Paulo, SP, Brazil.; 6 Universidade de São Paulo, Instituto de Ciências Biomédicas, Laboratório de Imunologia Humana, São Paulo, Brazil.

**Keywords:** COVID-19, host genetics, extreme phenotypes, infectious disease

## Abstract

COVID-19 comprises clinical outcomes of SARS-CoV-2 infection and is highly heterogeneous, ranging from asymptomatic individuals to deceased young adults without comorbidities. There is growing evidence that host genetics play an important role in COVID-19 severity, including inborn errors of immunity, age-related inflammation and immunosenescence. Here we present a brief review on the known order of events from infection to severe system-wide disturbance due to COVID-19 and summarize potential candidate genes and pathways. Finally, we propose a strategy of subject’s ascertainment based on phenotypic extremes to take part in genomic studies and elucidate intrinsic risk factors involved in COVID-19 severe outcomes.

Worldwide populations were significantly affected in 2020 due to the SARS-CoV-2 virus outbreak and individual’s health was heterogeneously affected regarding age, sex and comorbidities by the clinical manifestation of the infection (COVID-19). Up to 17% are severe cases of COVID-19 leading to hospitalization and ultimately causing up to 6% of death. Some patients require intensive care units (ICU), which faces overflowing occupancy during pandemic peaks (Garg *et al.*, 2020; Griffin, 2020). This balance between the relative and absolute number of individuals with severe symptoms and corresponding distribution of healthcare capacity is critical to prevent a sanitary crisis (Vergano *et al.*, 2020). When ICUs run in full occupancy, mild to severe cases that would eventually be treated and dismissed may progress and lead to worse outcomes than expected under normal occupancy conditions, not to mention overwhelming healthcare teams that care for patients with other conditions (Rosenbaum and Malina, 2020), exposure of staff, and disrupting protective equipment supply chain (Cavallo *et al.*, 2020). Therefore, predicting the proportion of individuals at higher risk of severity could improve healthcare management decisions and budget allocation by implementation of evidence-based public policies. Risk segmentation can be observed empirically by stratification of individuals based in age, sex, previous comorbidities, and markers (Zheng *et al.*, 2020). However, many risk factors are prevalent and the extent of population-based risk could be more precisely achieved by profiling individual’s genomic variability that is associated to increased risk, in a similar fashion of estimating carriers of pathogenic alleles linked to recessive disorders (Zhang *et al.*, 2019) or newborn screening for treatable monogenic conditions (Kelly *et al.*, 2016).

Even though comorbidities are undoubtedly risk factors in COVID-19 severe outcomes, the fact that a proportion of individuals with a healthy status, unaffected by prevalent chronic disorders associated with COVID-19 severity or young patients are progressing to severe complications, suggest a role of genetic susceptibility (Zhang *et al.*, 2020). Such risk profiles are likely to be concealed in the absence of challenging triggers, such as an infection. Indeed, inborn errors of immunity or immunodeficiencies that segregate in families are rare clinical phenotypes caused by highly penetrant variants in over 400 genes (Tangye *et al.*, 2020; Zhang *et al.*, 2020). In theory, even if combined, such monogenic manifestations are likely to explain, at most, a fraction of cases, of yet unknown magnitude. 

Given the rate of severe outcomes across the population strata, regardless of ancestries and admixture, and the wide heterogeneity of genes and pathways implicated in host-pathogen interactions, it is reasonable to assume a multifactorial mode of inheritance for susceptibility to COVID-19 progression. Such model, however, implies challenges to study design and analytical choices, since there is no prior knowledge of which cases might be explained by rare Mendelian inherited conditions or by a polygenic profile resulting from rare and common variants with individually small to moderate effect sizes. Modulation of risk by indirect genetic factors (e.g. causal factors of comorbidities) and environmental factors, such as exposure to variable viral loads (He *et al.*, 2020) or co-infections that may overwhelm shared systems with SARS-CoV-2 (Bradbury *et al.*, 2020), demonstrate the complexity in pinpointing susceptibility drivers (Figure 1).

**Figure 1 -:**
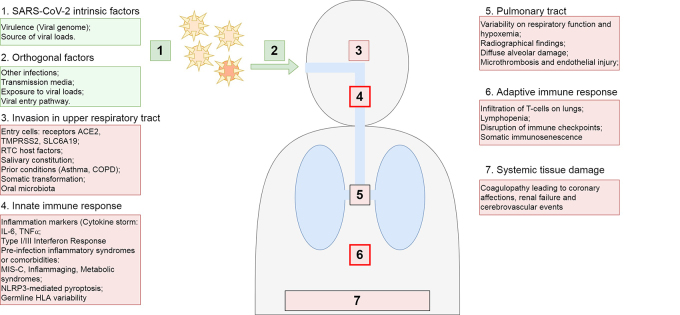
Factors driving SARS-CoV-2 infection and severity of COVID-19 outcomes. Non-host factors (green) and intrinsic host factors (pink) in seven hypothetical steps that may vary and result in different outcomes.

The wide variability of symptoms in individuals infected by SARS-COV-2 allows to hypothesize candidate genes and pathways that might play a role in risk of (and alternatively, in protection against) severity progression of COVID-19, harboring variation that alters function and/or expression with heterogeneous contribution to the combined individual profile. The X-linked *ACE2* gene encodes the Angiotensin-converting enzyme 2, which is expressed in many organs, including nasal and upper airway mucosa, interacts with SARS-CoV-2 spike proteins, followed by TMPRSS2-mediated spike processing and facilitation of viral entry into host cells (Wu and Zheng, 2020; Yan *et al.*, 2020). SLC6A19, although not co-expressed in the upper respiratory tract (Wu and Zheng, 2020), is mainly expressed in enterocytes and antagonizes TMPRSS2, and may play a role in gastrointestinal virion intake. TMPRSS2 is indeed key for viral fusion and considered a candidate for drug targets reducing viral entry (Catanzaro *et al.*, 2020). 

It is plausible that coding and regulatory variants in the genes encoding products associated to direct viral entry might influence susceptibility and protection against the first step of infection. It was hypothesized that the higher incidence of COVID-19 in men compared to women could be associated to X-linked factors (Gagliardi *et al.*, 2020). There is evidence for higher ACE2 expression in lungs in patients with comorbidities (Pinto *et al.*, 2020), but sex-specific data are still not available and the balance between hemizygous expression in men and local X-inactivation in women require further investigation. Recently, a sequencing study of Italian patients has suggested common and rare coding variants in ACE2 enriched in patients when compared to controls (Benetti *et al.*, 2020), although a larger number of samples would be ideal to provide supporting evidence. Other host factors involved in formation and activation of the replicase-transcription complex (RTC) are likely to influence success of initial cellular infection cycles (Baric *et al.*, 2008), among which some were detected with an in vitro proteomics approach (V'kovski *et al.*, 2019).

Once viral particles start replicating in host cells, activation of innate and adaptive immune response is expected under physiological conditions. Activating the innate immune response with exacerbated release of proinflammatory cytokines and chemokines (known as ‘cytokine storm’) has been associated with disease severity. This terminology is forgiving, since it accepts heterogeneous patterns of changes in mediator levels in attempt of fighting infection, ultimately being deleterious to host cells themselves (Tisoncik *et al.*, 2012). During the acute response, interleukin-6 (IL-6) is key and has been consistently observed in high levels among patients with severe COVID-19 outcomes and generic hyperinflammatory phenotype (Sinha *et al.*, 2020). Many pathways leading to viral stimulation of innate response and inflammation might play a role in SARS-CoV-2. The canonical and non-canonical inflammasome NLRP3-mediated pathways are candidates since they are sensitive to single stranded RNA viruses and induce pyroptosis fate on host cells (Hayward *et al.*, 2018), which in turn releases pro-inflammatory factors system-wide (Yap *et al.*, 2020). Indeed, the NLRP3 inflammasome is activated in response to SARS-CoV-2 infection, and inflammasome-derived products in sera were found to be correlated with COVID-19 severity (Rodrigues *et al.*, 2020).

The primary source of the exacerbated inflammation itself is still an open problem. If prior chronic inflammation is observed, would it drive the infection-mediated proinflammatory response to reach a certain systemic threshold? There are three major axes of empirical evidence that might support this hypothesis. First, children may be predisposed to hyperinflammatory responses. Cases of multisystem inflammatory syndrome in children (MIS-C) are being observed in severe COVID-19 worldwide (Dufort *et al.*, 2020; Feldstein *et al.*, 2020), an otherwise rare condition that is similar to Kawasaki syndrome, where children manifest inflammation in various systems. As an outcome, cardiovascular dysfunctions are observed, along with elevated levels of C-reactive protein (CRP), D-dimer and troponin, most of which are also observed in high levels in aged patients with severe COVID-19 (Li Q *et al.*, 2020a; Zhou *et al.*, 2020).

Second, the elevated baseline inflammatory status in elderly (‘Inflammaging’) is associated to frailty, morbidity and mortality regardless of infection (chronic low-grade sterile inflammation). Several factors are associated to this condition, but, at large, senescent cells, including lymphocytes, that secrete cytokines, chemokines and other pro-inflammatory appear to be involved in this phenotype (Akbar and Gilroy, 2020). Although somatic variation is likely to play a role in immunosenescence, it is observed that the distribution of age-related release of pro-inflammatory molecules is variable and long-lived individuals may escape the ‘inflammaging’ phenotype (Fulop *et al.*, 2017).

The last empirical evidence lies in the risk of comorbidities associated with severe COVID-19. Since the beginning of the pandemic, it has been reported that hypertension, obesity and diabetes (Guan *et al.*, 2020; Richardson *et al.*, 2020) are risk factors for critical COVID-19 outcomes, including death. Low-grade chronic inflammation is a common feature of obesity and metabolic syndromes, and its role in increasing the risk of complications due to SARS-CoV-2 infection cannot be excluded, deserving further investigation (Chiappetta *et al.*, 2020).

Regarding adaptive immunity, decrease of lymphocyte counts and sub-population distribution have also been correlated with worse COVID-19 progression, specifically reduced proportions of CD4+ and CD8+ as predictors of mortality and organ damage (Li D *et al.*, 2020b). There are potential causes for lymphopenia to be considered, including direct infection of lymphocytes, which require further confirmation due to the fact that these cells are likely to be ACE2-negative (Hamming *et al.*, 2004; Xu *et al.*, 2020). In addition, pathological dissections in post-mortem samples should confirm damage to lymphatic organs such as thymus and spleen, leading to lymphocytic dysfunction (Tan *et al.*, 2020). The above mentioned disruption of cytokines’ release, such as IL-6 and especially tumor necrosis factor (TNF)⍺, could down-regulate differentiation and may impact proliferation of exhausted T lymphocytes (Moro-Garcia *et al.*, 2018). Although this is still speculative, immunotherapy trials for COVID-19 by blocking immune checkpoints were suggested (Pickles *et al.*, 2020) and require further confirmation (Luo *et al.*, 2020). Lymphocytes can also be inhibited by circulating lactic acid (Chhetri *et al.*, 2020). Although considering metabolic acidosis as a risk factor during COVID-19 elevated cytokine release is still hypothetical, circulating lactate dehydrogenase (LDH) is consistently observed in patients with severe outcomes (Han *et al.*, 2020; Lu *et al.*, 2020) and might reflect system-wide cell death through apoptosis and pyroptosis (Rayamajhi *et al.*, 2013). Lastly, immunosenescence can be accelerated in elderly due to accumulation of somatic mutations (Zhang, Dong *et al.*, 2019).

The major mechanism of immune evasion of coronaviruses including SARS-CoV-2 is interference with multiple non-structural and accessory proteins of coronaviruses in the production of and response to type I (Hadjadj *et al.*, 2020) and type III interferons (Yuen *et al.*, 2020). Combined with the association of an early innate antiviral interferon response with mild (Park and Iwasaki, 2020), it is possible to anticipate that variants in genes associated with this pathway may render carriers refractory to productive COVID-19 infection. 

Once the infection is established on alveoli, progression to pneumonia, breathing difficulties, and hypoxemia lead patients to mechanical ventilatory support. Qualitative and quantitative thresholds on chest radiographic findings, mostly peripheral lung ground-glass opacities, are being used at the healthcare front as COVID-19 probable classification prior to RT-PCR confirmation, since clinical worsening can be steep (Shi *et al.*, 2020). Variability is observed among asymptomatic: even before classical symptoms appear, a proportion of individuals that tested positive had imaging findings (Meng *et al.*, 2020). Interestingly, lung abnormalities could be found in a case that wasn’t primarily aware of COVID-19 (Barajas *et al.*, 2020), although it is still early to estimate the rate of incidental findings.

There is, therefore, heterogeneity regarding the correlation of symptoms, lung damage and hypoxemia, which in turn can require interventions such as invasive mechanical ventilation. In an attempt to understand the most severe outcomes, autopsy studies were initiated in patients who died of COVID-19, in order to investigate pathological changes in several organs. Postmortem lung analysis identified diffuse alveolar damage in different stages of tissue injury and repair by fibroproliferation, and a high incidence of pulmonary embolism, which can account for up to a third of death causes (Deshpande, 2020; Duarte‐Neto et al., 2020). Microthrombosis in the alveolar tissue has been detected in up to 80% of the cases (Dolhnikoff et al 2020; Duarte‐Neto et al., 2020). Further investigation revealed infiltration of T-cells, severe endothelial injury, presence of intracellular virus and cell membrane disruption, with evidence of intussusceptive angiogenesis, suggesting a compensatory response (Ackermann *et al.*, 2020).

Overall, thromboembolic events in the lungs or elsewhere such as heart, kidneys and brain are likely causes of death in severe cases, indicating a probable role for vascular phenotypes, mainly clot formation and distribution (Wadman, 2020). Coagulopathy is supported by consistent findings of elevated D-dimer levels, which in combination with CRP levels, are predictive of critical illness and death from admission date and beyond (Al-Samkari *et al.*, 2020). Therefore, recovered survivors from severe complications due to COVID-19 are likely to have overcome most vascular phenotypes but may have persistent symptoms due to damaged lungs (Carfì *et al.*, 2020) and neurological features, particularly encephalomyelitis (Paterson *et al.*, 2020).

The sequence of observed events and description of general endophenotypes from infection to death or recovery, along the spectrum of COVID-19 progression raises concerns on how to define and select groups of individuals that would capture underlying causality signals, particularly in complex phenotypes with likely multifactorial inheritance. It has been previously proposed that extreme phenotypes might improve the ability of identifying variants and genes ranked with larger effect sizes (Pérez-Gracia *et al.*, 2010; Peloso *et al.*, 2016). This can be achieved by selecting groups with well-defined phenotypes from the extremes of the distribution, and performing rare-variant association studies such as burden analyses, where collapsing variants into biologically relevant sets, usually genes and pathways, provide signals of association even in smaller sample sizes (Lee *et al.*, 2014). A similar collection strategy in COVID-19 has been proposed by expert groups in immunodeficiency research (Casanova *et al.*, 2020). 

Considering the extent of SARS-CoV-2 infection and COVID-19 heterogeneity in outcomes worldwide, retrospective classification and re-grouping is already being applied by strategies of meta-analyses. Regarding power estimates when using extreme sampling as an optimization strategy, Peloso et al. (2016) performed simulations using real data of HDL-C distributions and a combination of rare and common variants in *ABCA1* gene and provided evidence that smaller sample sizes are required to achieve comparable power on common variants with reduced effect sizes, trading off with some level of overestimation of the proportion of functional variants. If selection threshold is stricter, likelihood of power gains due to extreme selection increases. It has been advocated that since rare variants have a wider effect size range, sequencing studies with burden strategies can benefit from extreme sampling (Peloso *et al.*, 2016; Barnett *et al.*, 2013).

We propose to enrich groups of individuals that have severe lethal outcome with others that remain asymptomatic, ideally against the odds of commonly observed features, such as age and distribution of comorbidities (Figure 2). 

**Figure 2 -:**
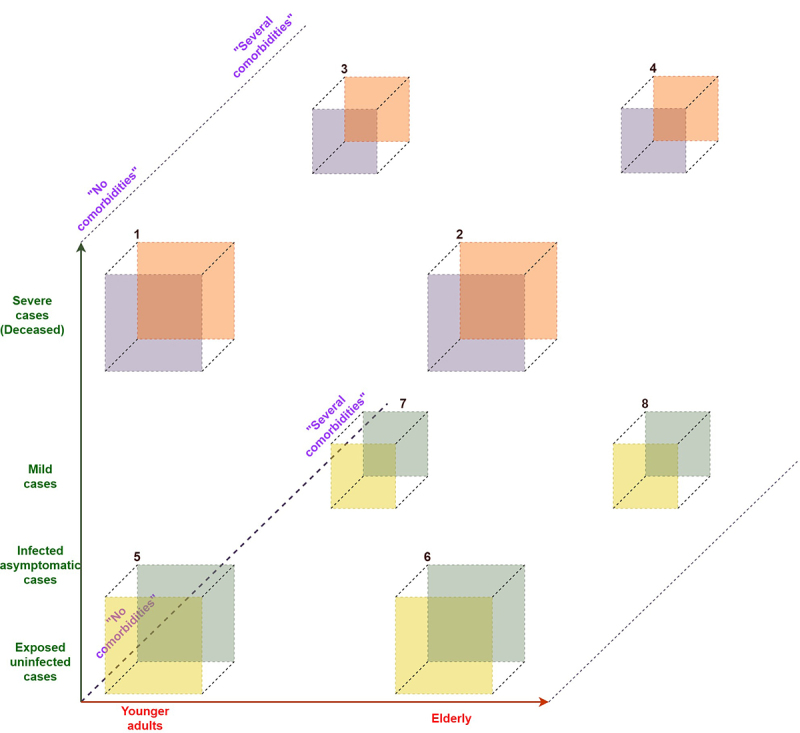
Hypothetical distribution of extreme COVID-19 phenotypes per generalized risk groups. Groups 1-4 comprise the most susceptible ends. Group 1 opposes to Group 8, from most susceptible to most resistant. Individuals below 1-4 groups are expected to have severe outcomes but not leading to death, and compose a ‘resilient’ phenotype towards the corner below group 4 (elderly, with several comorbidities that were recovered).

Considering the phase of contamination itself to be partially explained by genetics, we have initiated a collection of exposed unaffected individuals. Since controlling for exposure is challenging, our rationale involved the collection of couples or individuals from the same household that are discordant for symptoms and COVID-19 confirmation (one positive and one negative). Special attention will be given to concordance or discordance in twins, monozygotic as compared to dizygotic twins, even though it is unlikely to identify adult twins co-living (fulfilment of exposure control). Asymptomatic exposed individuals who test negative for viremia and antibodies are named ‘Resistant’. Since exposure levels might determine an infection event after initial sampling and that the viral strains are likely to be the same within each pair of individuals, additional confirmation of negative infection after periods of time will support ‘Resistant’ phenotype, or alternatively reclassify these individuals as ‘Susceptible’ under a different context.

As a second group, we began collecting samples from patients who died of COVID-19 and underwent autopsy procedure classified as a ‘Susceptible’ group. Retrospective collection of presence of comorbidities and estimating evidences of *ante-mortem* versus *post-mortem* vascular pathologies may provide a glimpse of prior risk and injuries due to SARS-CoV-2 infection itself. 

Third, a collection of recovered nonagenarians and centenarians might provide insights on compensatory effects of system-wide resilience (named ‘Resilience’ group), which are likely to result from a combination of effective immune response, reduced levels of inflammation and protection from coagulopathies. In this stage, we aim at collecting DNA, RNA and peripheral blood mononuclear cells from individuals admitted to a hospital and recollecting after discharge, providing additional molecular phenotypes to improve filtering variants within genes and pathways observed to be altered. 

This ongoing project (IRB approval, CEP IB-USP CAAE 34786620.2.0000.5464) has to date collected clinical data and biological samples from 380 individuals, most of which have been already whole-exome sequenced, RNA and vials of peripheral blood mononucleate cells (PBMCs) from most participants stored for downstream functional analyses.

Genome-wide association studies (GWAS) with case-control design in COVID-19 started to yield results in Spanish and Italian individuals. They have detected association signals in a locus at 3p21.31 comprising interesting candidates such as genes encoding SIT1, an interactor with ACE2, and CXCR6, a regulator of memory CD8 T cells (Ellinghaus *et al.*, 2020). ABO locus was also detected, overlapping with a clinical suggestion of specific blood types being enriched in severe groups, although this is still controversial (Latz *et al.*, 2020; Wu *et al.*, 2020). The authors stress the need for ascertainment of controls, since dynamics of infection and variable symptomatology might compromise stratification. 

Traditional GWAS using common variants may not capture the strongest association signals, since genetic heterogeneity across groups of patients and controls may promote diverse and complex genomic architectures. Including rare variation obtained by sequencing whole exomes or, ideally, whole genomes, followed by burden analyses where rare variants are collapsed per gene or pathway might increase the ability to detect signals (Lanktree *et al.*, 2010; Peloso *et al.*, 2016; Xu *et al.*, 2018). Selection and characterization of extreme phenotypes in response to HIV-1 infection led to discovery of the 32-base pair deletion in the chemokine receptor 5 (*CCR5Δ32*) (Dean *et al.*, 1996), which helped elucidation of pathways associated to AIDS. Other studies on reduced penetrance within families, that is, extreme phenotypes co-segregating with pathogenic mutation carriers in the same pedigree, can provide clues for modifier genes (Cooper *et al.*, 2013), including those implicated in variable expressivity, for instance compensating the absence of key protein in muscle physiology (Vieira *et al.*, 2015). Differential cellular susceptibility to infection was also verified in twins exposed to Zika virus (Caires-Júnior *et al.*, 2018), suggesting that discordant outcomes can provide insights about the intrinsic factors driving host-pathogen interactions.

We hypothesize that using extreme phenotypes of properly ascertained groups will improve aggregation of variants with larger effect size, which, in combination, will allow polygenic risk stratification. Collaborative initiatives such as the COVID Human Genetic Effort (Zhang *et al.*, 2020) and the COVID-19 Host Genetics Initiative (2020) were organized to systematically aggregate data across different regions worldwide. Since the etiology of COVID-19 host susceptibility and protection is likely to be multifactorial, it is expected that a large number of samples will be needed in order to identify loci that associate with corresponding phenotypes, therefore collaboration is critical. We emphasize the need for including admixed individuals with ancestries that go beyond European, which would not only contribute to reduce health disparities when describing the particularities of severe outcomes on diverse populations, but may provide insights from genes and pathways that harbor specific variants associated to phenotypes of interest such as severe outcomes of COVID-19. 
